# *E*-Notopterol

**DOI:** 10.1107/S1600536809005030

**Published:** 2009-02-18

**Authors:** Andreas Schinkovitz, Ferdinand Belaj, Olaf Kunert, Rudolf Bauer

**Affiliations:** a Institute of Pharmaceutical Sciences, Department of Pharmacognosy, Karl-Franzens University Graz, Universitätsplatz 4, A-8010 Graz, Austria; b Institute of Chemistry, Karl-Franzens University Graz, Schubertstrasse 1, A-8010 Graz, Austria; c Institute of Pharmaceutical Sciences, Department of Pharmaceutical Chemistry, Karl-Franzens University Graz, Universitätsplatz 1, A-8010 Graz, Austria

## Abstract

The title compound (systematic name: 4-{[(2*E*)-5-hydr­oxy-3,7-dimethylocta-2,6-dien-1-yl]­oxy}-7*H*-furo[3,2-*g*][1]benzopyran-7-one), C_21_H_22_O_5_, is a known furan­ocoumarin, which was isolated from the Chinese herbal product Radix seu Rhizoma Notopterygii. The crystal structure shows a weak O—H⋯O hydrogen bond.

## Related literature

For the isolation, see: Yang *et al.* (1994 ▶); Xiao *et al.* (1994 ▶). For NMR shifts and coupling constants of related compounds, see: Hasegawa *et al.* (1999 ▶); Chemical Abstract Service (2009 ▶). 
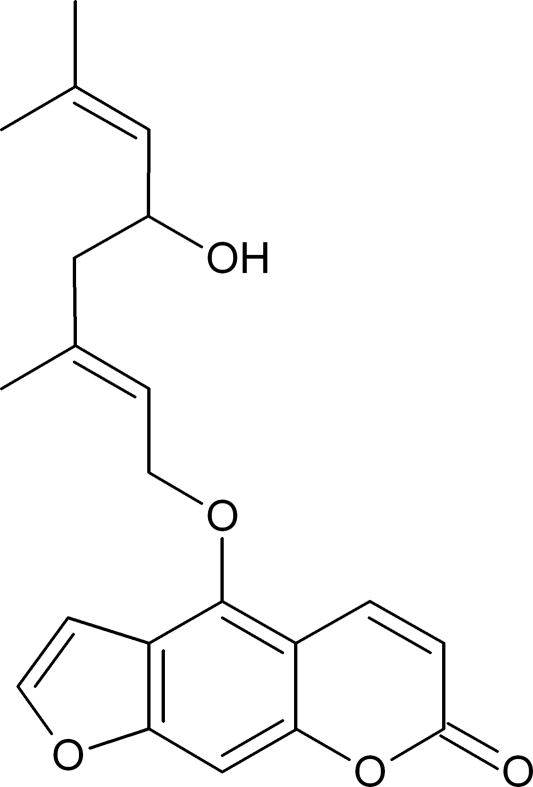


## Experimental

### 

#### Crystal data

C_21_H_22_O_5_
*M* *_r_* = 354.39
Triclinic, 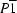

*a* = 6.4317 (10) Å
*b* = 8.0912 (16) Å
*c* = 17.206 (3) Å
α = 91.802 (15)°
β = 94.240 (13)°
γ = 97.473 (15)°
*V* = 884.6 (3) Å^3^
*Z* = 2
Mo *K*α radiation
μ = 0.09 mm^−1^
*T* = 95 K
0.48 × 0.44 × 0.32 mm

#### Data collection

Stoe four-circle diffractometer
Absorption correction: none
4234 measured reflections
3462 independent reflections
2941 reflections with *I* > 2σ(*I*)
*R*_int_ = 0.023
3 standard reflections every 100 reflections intensity decay: 0.4%

#### Refinement

*R*[*F* ^2^ > 2σ(*F* ^2^)] = 0.043
*wR*(*F* ^2^) = 0.113
*S* = 1.04
3462 reflections
249 parameters
Only H-atom displacement parameters refined
Δρ_max_ = 0.38 e Å^−3^
Δρ_min_ = −0.21 e Å^−3^

### 

Data collection: local software; cell refinement: local software; data reduction: local software; program(s) used to solve structure: *SHELXS97* (Sheldrick, 2008 ▶); program(s) used to refine structure: *SHELXL97* (Sheldrick, 2008 ▶); molecular graphics: modified *ORTEP* (Johnson, 1965 ▶); software used to prepare material for publication: *SHELXL97*.

## Supplementary Material

Crystal structure: contains datablocks global, I. DOI: 10.1107/S1600536809005030/bt2871sup1.cif

Structure factors: contains datablocks I. DOI: 10.1107/S1600536809005030/bt2871Isup2.hkl

Additional supplementary materials:  crystallographic information; 3D view; checkCIF report

## Figures and Tables

**Table 1:** Hydrogen-bond geometry (Å, °)

*D*—H⋯*A*	*D*—H	H⋯*A*	*D*⋯*A*	*D*—H⋯*A*
O5—H51⋯O8^i^	0.84	2.57	3.2193 (16)	135

Symmetry code: (i) 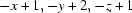.

**Fig. 1.**
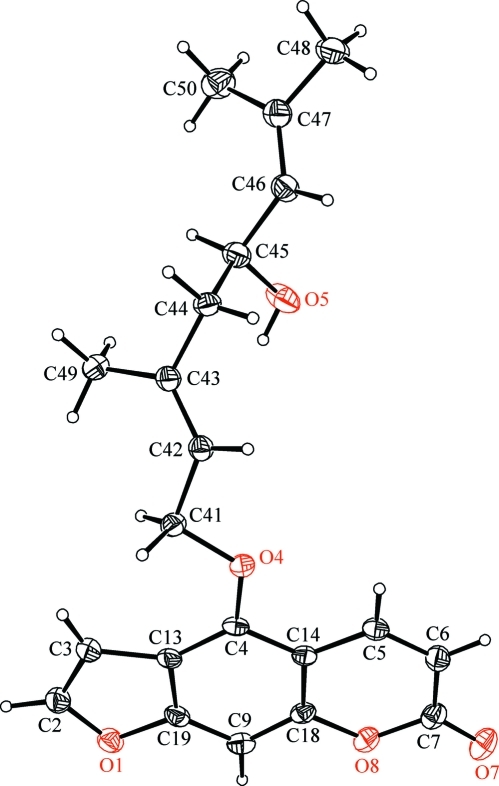
ORTEP plot (Johnson, 1965) showing the atomic numbering scheme. The probability ellipsoids are drawn at the 50% probability level.

**Fig. 2.**
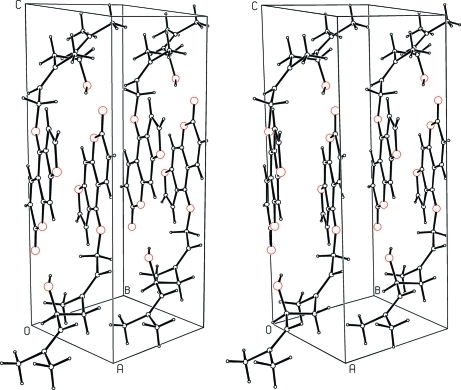
Stereoscopic ORTEP plot (Johnson, 1965) of the packing. The atoms are drawn with arbitrary radii.

## supplementary crystallographic information

### Comment

Rhizoma seu Radix Notopterygii is a herbal preparation commonly used in traditional Chinese medicine. It may either contain roots or rhizomes of *N. incisum* or *N. forbesii* (Apiaceae) or a mixture of both plants. The isolation of *E*-Notopterol (Fig.1) has been previously reported for both species (Yang *et al.*, 1994; Xiao *et al.* 1994), which naturally occur in the region of South Central Asia, but the crystal structure has not been reported until now. In the process of structure elucidation, the title compound was primarily identified by ^1^H and ^13^C NMR experiments. Observed chemical shifts and coupling constants were in compliance with previously published literature (Hasegawa *et al.*, 1999; Chemical Abstract Service, 2009). Further support was provided by the ESI-MS analysis, indicating the pseudo molecular ion at 377.29 m/*z* [M—Na^+^].

The crystal structure analysis   confirmed the proposed structure as 4-{[(2*E*)-5-hydroxy-3,7-dimethyl- octa-2,6-dien-1-yl]oxy}-7*H*-furo[3,2-*g*][1]benzopyran-7-one. All the atoms are lying on general positions. In Fig. 1 the S-enantiomer of the racemate is shown. The least-squares plane through the non-H atoms around the double bond C42=C43 enclose an angle of 12.36 (9)° with the plane through the atoms around C46=C47, and further an angle of 41.35 (6)° with the plane through the tricycle. The packing is dominated by an antiparallel stacking of the tricycles (s. Fig. 2) preventing the formation of a strong hydrogen bond of the OH group [O5—H51···.O8^i^ 134.5°, O5···.O8^i^ 3.2193 (16) Å; symmetry transformation used to generate the equivalent atom: (i) 1 - *x*, 2 - *y*, 1 - *z*].

### Experimental

Plant Material: Rhizoma seu Radix Notopterygii was purchased from Plantasia (Oberndorf, Austria).

Isolation and purification: Plant material (2000 g) was grinded and extracted with 11 *L* dichloromethane at room temperature, yielding 381.87 g of dried extract after vacuum evaporation. Next 100 g dried extract were fractionated on a Silicagel 60 column (230–400 mesh, Merck, Darmstadt, Germany; size: 8.5 *x* 20 cm). In the process a stepwise gradient elution starting from 100% hexane (Hex) and going to 100% ethylacetate (EtOAc) was performed. For each step (2400 ml) the content of Hex in the eluent was reduced by 16.7 vol.%, and 6–8 subfractions were collected. Subfraction D7 collected between 2030 ml and 2400 ml from eluent D (Hex:EtOAc: 50:50, v:v) yielded in the isolation of 86.49 mg crystallized 1. Finally the colourless block crystals were washed with eluent C (Hex:EtOAc: 67:33, v:v) before being analyzed.

NMR analysis: ^1^H and ^13^C NMR spectra were recorded at 293 K on a Varian UnityInova 400 NMR spectrometer with a 5 mm broadband probe. E-Notpterol was dissolved in chloroform-*d* (Aldrich, USA) and TMS was used as internal standard.

NMR results: ^1^H NMR (CDCl_3_, 400 MHz) δ 1.70 (3*H*, d, J = 1.2, H-50), 1.72 (3*H*, d, J = 1.2, H-48), 1.77 (3*H*, s, H-49), 2.22 (1*H*, dd, J = 13.7, 5.0, H-44*b*), 2.31 (1*H*, dd, J = 13.7, 8.2, H-44*a*), 4.52 (1*H*, m, H-45), 4.98 (2*H*, d, J = 6.9, H-41), 5.18 (1*H*, pseudo td, J = 8.4, 1.3, H-46), 5.65 (1*H*, pseudo dt, J = 6.7, 1.0, H-42), 6.28 (1*H*, d, J = 9.8, H-6), 6.98 (1*H*, dd, J = 2.4, 0.8, H-3), 7.16 (1*H*, s, H-9), 7.60 (1*H*, d, J = 2.4, H-2), 8.16 (1*H*, d, J = 9.8, H-5); ^13^C NMR (CDCl_3_, 400 MHz) δ 17.0 (CH3, C-49), 18.2 (CH3, C-50), 25.7 (CH3, C-48), 47.7 (CH2, C-44), 66.4 (CH, C-45), 69.4 (CH2, C-41), 94.3 (CH, C-9), 105.0 (CH, C-3), 107.4 (C, C-14), 112.6 (CH, C-6), 114.0 (C, C-13), 122.4 (CH, C-42), 127.3 (CH, C-46), 135.6 (C, C-47), 139.5 (C, C-43), 139.5 (CH, C-5), 145.0 (CH, C-2), 148.7 (C, C-4), 152.6 (C, C-18), 158.1 (C, C-19), 161.3 (C, C-7).

LC—MS-analysis: Experiments were performed on a Thermo Finnigan Surveyor liquid chromatograph interfaced with a LCQ^TM^ Deca *XP*^PLUS^ mass detector. E-Notopterol was dissolved in methanol (HPLC grade, Merck, Darmstadt, Germany) and analyzed in the ESI+ mode under following conditions: Sheath gas flow: 70 units, ionization voltage: 5,50 kV, capillary voltage 15 V, tube lens offset: 50 V, capillary temperature: 623 K.

### Refinement

H atoms were located in a difference map, but geometrically positioned with C_aromatic_-H = 0.95Å, C_methyl_-H = 0.98Å, C_methylene_-H = 0.99Å and O-H = 0.84Å. The torsion angle about the C-O bond was also refined. The U values of the H atoms were refined using the same U values for similar H atoms.

### Crystal data

**Table:**

C_21_H_22_O_5_	*Z* = 2
*M**_r_* = 354.39	*F*(000) = 376
Triclinic, *P*1	*D*_x_ = 1.330 Mg m^−^^3^
Hall symbol: -P 1	Mo *K*α radiation, λ = 0.71069 Å
*a* = 6.4317 (10) Å	Cell parameters from 60 reflections
*b* = 8.0912 (16) Å	θ = 16.0–19.8°
*c* = 17.206 (3) Å	µ = 0.09 mm^−^^1^
α = 91.802 (15)°	*T* = 95 K
β = 94.240 (13)°	Block, colourless
γ = 97.473 (15)°	0.48 × 0.44 × 0.32 mm
*V* = 884.6 (3) Å^3^	

### Data collection

**Table:**

Stoe four-circle diffractometer	*R*_int_ = 0.023
Radiation source: fine-focus sealed tube	θ_max_ = 26.0°, θ_min_ = 2.5°
graphite	*h* = −7→7
ω–2θ scans	*k* = −9→1
4234 measured reflections	*l* = −21→21
3462 independent reflections	3 standard reflections every 100 reflections
2941 reflections with *I* > 2σ(*I*)	intensity decay: 0.4%

### Refinement

**Table:**

Refinement on *F*^2^	Primary atom site location: structure-invariant direct methods
Least-squares matrix: full	Secondary atom site location: difference Fourier map
*R*[*F*^2^ > 2σ(*F*^2^)] = 0.043	Hydrogen site location: inferred from neighbouring sites
*wR*(*F*^2^) = 0.113	Only H-atom displacement parameters refined
*S* = 1.04	*w* = 1/[σ^2^(*F*_o_^2^) + (0.0482*P*)^2^ + 0.2588*P*] where *P* = (*F*_o_^2^ + 2*F*_c_^2^)/3
3462 reflections	(Δ/σ)_max_ < 0.001
249 parameters	Δρ_max_ = 0.38 e Å^−^^3^
0 restraints	Δρ_min_ = −0.21 e Å^−^^3^

### Special details

**Table:**

Geometry. All e.s.d.'s (except the e.s.d. in the dihedral angle between two l.s. planes) are estimated using the full covariance matrix. The cell e.s.d.'s are taken into account individually in the estimation of e.s.d.'s in distances, angles and torsion angles; correlations between e.s.d.'s in cell parameters are only used when they are defined by crystal symmetry. An approximate (isotropic) treatment of cell e.s.d.'s is used for estimating e.s.d.'s involving l.s. planes.
Refinement. Refinement of *F*^2^ against ALL reflections. The weighted *R*-factor *wR* and goodness of fit *S* are based on *F*^2^, conventional *R*-factors *R* are based on *F*, with *F* set to zero for negative *F*^2^. The threshold expression of *F*^2^ > σ(*F*^2^) is used only for calculating *R*-factors(gt) *etc*. and is not relevant to the choice of reflections for refinement. *R*-factors based on *F*^2^ are statistically about twice as large as those based on *F*, and *R*- factors based on ALL data will be even larger.The non-hydrogen atoms were refined with anisotropic displacement parameters without any constraints. The H atoms of the 7*H*-Furo[3,2-*g*][1]benzopyran-7-one ring system were put at the external bisector of the C—C—C angle at a C—H distance of 0.95 Å and a common isotropic displacement parameter was refined (AFIX 43 of *SHELXL97*). The H atoms of the CH_2_ groups were refined with common isotropic displacement parameters for the H atoms of the same group and idealized geometry with approximately tetrahedral angles and C—H distances of 0.99 Å (AFIX 23 of *SHELXL97*). The H atoms bonded to a C atom of a C=C double bond were put at the external bisector of the C—C—C angle at a C—H distance of 0.95 Å but the individual isotropic displacement parameters are free to refine (AFIX 43 of *SHELXL97*). The H atom of the tertiary C—H group was refined with an individual isotropic displacement parameter and all *X*—C—H angles equal at a C—H distance of 1.00 Å (AFIX 13 of *SHELXL97*). The H atoms of the methyl groups were refined with common isotropic displacement parameters for the H atoms of the same group and idealized geometry with tetrahedral angles, enabling rotation around the *X*—C bond, and C—H distances of 0.98 Å (AFIX 137 of *SHELXL97*). The H atom of the OH group was refined with a tetrahedral C—O—H angle, enabling rotation around the C—O bond, O—H distance of 0.84 Å, and with an individual isotropic displacement parameter (AFIX 147 of *SHELXL97*).

### Fractional atomic coordinates and isotropic or equivalent isotropic displacement parameters (Å^2^)

**Table:**

	*x*	*y*	*z*	*U*_iso_*/*U*_eq_	
O1	0.08010 (16)	1.43591 (14)	0.41654 (6)	0.0244 (3)	
C2	0.0927 (2)	1.43677 (19)	0.33658 (9)	0.0246 (3)	
H2	−0.0037	1.4827	0.3016	0.028 (2)*	
C3	0.2568 (2)	1.36524 (19)	0.31406 (9)	0.0226 (3)	
H3	0.2957	1.3524	0.2622	0.028 (2)*	
C4	0.5403 (2)	1.23232 (17)	0.40535 (8)	0.0177 (3)	
C5	0.7767 (2)	1.13743 (18)	0.51262 (9)	0.0202 (3)	
H5	0.8670	1.1018	0.4760	0.028 (2)*	
C6	0.8224 (2)	1.11882 (19)	0.58937 (9)	0.0225 (3)	
H6	0.9445	1.0707	0.6059	0.028 (2)*	
C7	0.6889 (2)	1.17088 (19)	0.64707 (9)	0.0228 (3)	
O7	0.71375 (18)	1.15883 (16)	0.71723 (6)	0.0305 (3)	
O8	0.51411 (17)	1.24050 (14)	0.62003 (6)	0.0231 (3)	
C9	0.2897 (2)	1.33880 (19)	0.52396 (9)	0.0216 (3)	
H9	0.2045	1.3732	0.5626	0.028 (2)*	
C13	0.3631 (2)	1.31129 (18)	0.38445 (8)	0.0185 (3)	
C14	0.5942 (2)	1.21012 (17)	0.48527 (8)	0.0179 (3)	
C18	0.4657 (2)	1.26325 (18)	0.54174 (8)	0.0189 (3)	
C19	0.2464 (2)	1.36067 (18)	0.44558 (9)	0.0200 (3)	
O4	0.66912 (16)	1.17002 (14)	0.35580 (6)	0.0223 (3)	
C41	0.6201 (2)	1.17328 (19)	0.27226 (8)	0.0206 (3)	
H411	0.6516	1.2880	0.2540	0.022 (3)*	
H412	0.4695	1.1328	0.2584	0.022 (3)*	
C42	0.7567 (2)	1.05985 (18)	0.23652 (8)	0.0197 (3)	
H42	0.8956	1.0633	0.2601	0.030 (5)*	
C43	0.7031 (2)	0.95436 (18)	0.17484 (8)	0.0198 (3)	
C44	0.8569 (2)	0.84090 (19)	0.14954 (9)	0.0215 (3)	
H441	0.9936	0.8725	0.1801	0.031 (3)*	
H442	0.8797	0.8576	0.0939	0.031 (3)*	
C45	0.7817 (3)	0.6553 (2)	0.16021 (9)	0.0247 (4)	
H45	0.6569	0.6179	0.1228	0.026 (4)*	
O5	0.7265 (2)	0.62481 (16)	0.23818 (7)	0.0342 (3)	
H51	0.6537	0.6973	0.2528	0.068 (8)*	
C46	0.9538 (3)	0.5518 (2)	0.14538 (9)	0.0246 (3)	
H46	1.0863	0.5869	0.1731	0.022 (4)*	
C47	0.9430 (3)	0.4165 (2)	0.09793 (9)	0.0251 (3)	
C48	1.1328 (3)	0.3278 (2)	0.08976 (10)	0.0322 (4)	
H481	1.2543	0.3891	0.1207	0.042 (3)*	
H482	1.1628	0.3221	0.0348	0.042 (3)*	
H483	1.1048	0.2146	0.1085	0.042 (3)*	
C49	0.4927 (3)	0.9353 (2)	0.12808 (9)	0.0248 (3)	
H491	0.4120	1.0230	0.1448	0.043 (3)*	
H492	0.4150	0.8258	0.1365	0.043 (3)*	
H493	0.5146	0.9448	0.0725	0.043 (3)*	
C50	0.7499 (3)	0.3374 (2)	0.04907 (10)	0.0335 (4)	
H501	0.6298	0.3945	0.0608	0.058 (4)*	
H502	0.7204	0.2194	0.0609	0.058 (4)*	
H503	0.7737	0.3470	−0.0063	0.058 (4)*	

### Atomic displacement parameters (Å^2^)

**Table:**

	*U*^11^	*U*^22^	*U*^33^	*U*^12^	*U*^13^	*U*^23^
O1	0.0215 (5)	0.0223 (6)	0.0295 (6)	0.0061 (4)	−0.0006 (4)	−0.0023 (5)
C2	0.0264 (8)	0.0193 (8)	0.0272 (8)	0.0028 (6)	−0.0037 (6)	0.0002 (6)
C3	0.0268 (8)	0.0172 (7)	0.0236 (8)	0.0043 (6)	−0.0013 (6)	−0.0002 (6)
C4	0.0202 (7)	0.0113 (7)	0.0212 (7)	0.0004 (6)	0.0036 (6)	−0.0027 (6)
C5	0.0224 (8)	0.0142 (7)	0.0234 (8)	0.0002 (6)	0.0025 (6)	−0.0003 (6)
C6	0.0241 (8)	0.0178 (7)	0.0247 (8)	0.0007 (6)	−0.0012 (6)	0.0025 (6)
C7	0.0239 (8)	0.0191 (8)	0.0227 (8)	−0.0053 (6)	−0.0015 (6)	0.0015 (6)
O7	0.0328 (6)	0.0363 (7)	0.0199 (6)	−0.0044 (5)	−0.0006 (5)	0.0032 (5)
O8	0.0251 (6)	0.0251 (6)	0.0183 (5)	0.0006 (5)	0.0026 (4)	−0.0005 (4)
C9	0.0199 (7)	0.0190 (8)	0.0254 (8)	−0.0001 (6)	0.0053 (6)	−0.0041 (6)
C13	0.0212 (7)	0.0118 (7)	0.0217 (7)	0.0011 (6)	0.0005 (6)	−0.0018 (6)
C14	0.0201 (7)	0.0120 (7)	0.0208 (7)	−0.0001 (6)	0.0014 (6)	−0.0016 (6)
C18	0.0224 (7)	0.0144 (7)	0.0185 (7)	−0.0026 (6)	0.0014 (6)	−0.0013 (6)
C19	0.0180 (7)	0.0131 (7)	0.0283 (8)	0.0016 (6)	0.0001 (6)	−0.0014 (6)
O4	0.0263 (6)	0.0255 (6)	0.0168 (5)	0.0100 (5)	0.0020 (4)	−0.0023 (4)
C41	0.0266 (8)	0.0184 (7)	0.0167 (7)	0.0033 (6)	0.0014 (6)	0.0002 (6)
C42	0.0217 (7)	0.0186 (7)	0.0192 (7)	0.0032 (6)	0.0022 (6)	0.0020 (6)
C43	0.0255 (8)	0.0163 (7)	0.0181 (7)	0.0032 (6)	0.0037 (6)	0.0038 (6)
C44	0.0277 (8)	0.0195 (8)	0.0180 (7)	0.0043 (6)	0.0043 (6)	−0.0008 (6)
C45	0.0281 (8)	0.0212 (8)	0.0256 (8)	0.0058 (7)	0.0039 (6)	0.0000 (6)
O5	0.0427 (7)	0.0292 (7)	0.0366 (7)	0.0163 (6)	0.0186 (6)	0.0099 (5)
C46	0.0267 (8)	0.0224 (8)	0.0253 (8)	0.0058 (7)	0.0028 (6)	0.0017 (6)
C47	0.0329 (9)	0.0202 (8)	0.0233 (8)	0.0049 (7)	0.0062 (6)	0.0053 (6)
C48	0.0444 (10)	0.0266 (9)	0.0290 (9)	0.0141 (8)	0.0090 (7)	0.0005 (7)
C49	0.0306 (9)	0.0237 (8)	0.0200 (7)	0.0047 (7)	−0.0004 (6)	−0.0013 (6)
C50	0.0424 (10)	0.0243 (9)	0.0322 (9)	−0.0008 (8)	0.0030 (8)	−0.0013 (7)

### Geometric parameters (Å, °)

**Table:**

O1—C19	1.3688 (18)	C41—H412	0.99
O1—C2	1.3843 (19)	C42—C43	1.336 (2)
C2—C3	1.343 (2)	C42—H42	0.95
C2—H2	0.95	C43—C49	1.509 (2)
C3—C13	1.454 (2)	C43—C44	1.512 (2)
C3—H3	0.95	C44—C45	1.537 (2)
C4—O4	1.3613 (18)	C44—H441	0.99
C4—C13	1.407 (2)	C44—H442	0.99
C4—C14	1.416 (2)	C45—O5	1.4336 (19)
C5—C6	1.349 (2)	C45—C46	1.504 (2)
C5—C14	1.436 (2)	C45—H45	1.00
C5—H5	0.95	O5—H51	0.84
C6—C7	1.447 (2)	C46—C47	1.337 (2)
C6—H6	0.95	C46—H46	0.95
C7—O7	1.2145 (19)	C47—C50	1.504 (2)
C7—O8	1.3788 (19)	C47—C48	1.507 (2)
O8—C18	1.3834 (18)	C48—H481	0.98
C9—C18	1.375 (2)	C48—H482	0.98
C9—C19	1.378 (2)	C48—H483	0.98
C9—H9	0.95	C49—H491	0.98
C13—C19	1.413 (2)	C49—H492	0.98
C14—C18	1.413 (2)	C49—H493	0.98
O4—C41	1.4504 (17)	C50—H501	0.98
C41—C42	1.499 (2)	C50—H502	0.98
C41—H411	0.99	C50—H503	0.98
			
C19—O1—C2	105.94 (12)	C43—C42—H42	116.7
C3—C2—O1	112.26 (14)	C41—C42—H42	116.7
C3—C2—H2	123.9	C42—C43—C49	124.47 (14)
O1—C2—H2	123.9	C42—C43—C44	119.56 (14)
C2—C3—C13	106.62 (14)	C49—C43—C44	115.95 (13)
C2—C3—H3	126.7	C43—C44—C45	113.08 (13)
C13—C3—H3	126.7	C43—C44—H441	109.0
O4—C4—C13	126.59 (13)	C45—C44—H441	109.0
O4—C4—C14	114.47 (13)	C43—C44—H442	109.0
C13—C4—C14	118.94 (13)	C45—C44—H442	109.0
C6—C5—C14	121.15 (14)	H441—C44—H442	107.8
C6—C5—H5	119.4	O5—C45—C46	106.58 (13)
C14—C5—H5	119.4	O5—C45—C44	111.88 (13)
C5—C6—C7	121.23 (15)	C46—C45—C44	110.45 (13)
C5—C6—H6	119.4	O5—C45—H45	109.3
C7—C6—H6	119.4	C46—C45—H45	109.3
O7—C7—O8	116.20 (14)	C44—C45—H45	109.3
O7—C7—C6	126.77 (15)	C45—O5—H51	109.5
O8—C7—C6	117.03 (13)	C47—C46—C45	127.87 (15)
C7—O8—C18	122.82 (12)	C47—C46—H46	116.1
C18—C9—C19	114.72 (14)	C45—C46—H46	116.1
C18—C9—H9	122.6	C46—C47—C50	125.44 (16)
C19—C9—H9	122.6	C46—C47—C48	120.82 (15)
C4—C13—C19	117.12 (13)	C50—C47—C48	113.73 (15)
C4—C13—C3	138.18 (14)	C47—C48—H481	109.5
C19—C13—C3	104.68 (13)	C47—C48—H482	109.5
C18—C14—C4	119.34 (13)	H481—C48—H482	109.5
C18—C14—C5	117.56 (13)	C47—C48—H483	109.5
C4—C14—C5	123.09 (13)	H481—C48—H483	109.5
C9—C18—O8	116.11 (13)	H482—C48—H483	109.5
C9—C18—C14	123.71 (14)	C43—C49—H491	109.5
O8—C18—C14	120.18 (13)	C43—C49—H492	109.5
O1—C19—C9	123.36 (14)	H491—C49—H492	109.5
O1—C19—C13	110.49 (13)	C43—C49—H493	109.5
C9—C19—C13	126.15 (14)	H491—C49—H493	109.5
C4—O4—C41	119.53 (11)	H492—C49—H493	109.5
O4—C41—C42	105.47 (12)	C47—C50—H501	109.5
O4—C41—H411	110.6	C47—C50—H502	109.5
C42—C41—H411	110.6	H501—C50—H502	109.5
O4—C41—H412	110.6	C47—C50—H503	109.5
C42—C41—H412	110.6	H501—C50—H503	109.5
H411—C41—H412	108.8	H502—C50—H503	109.5
C43—C42—C41	126.62 (14)		
			
C19—O1—C2—C3	0.41 (17)	C4—C14—C18—O8	179.09 (12)
O1—C2—C3—C13	0.19 (18)	C5—C14—C18—O8	−1.7 (2)
C14—C5—C6—C7	0.2 (2)	C2—O1—C19—C9	178.89 (14)
C5—C6—C7—O7	179.33 (15)	C2—O1—C19—C13	−0.87 (16)
C5—C6—C7—O8	−0.2 (2)	C18—C9—C19—O1	−178.88 (13)
O7—C7—O8—C18	179.60 (13)	C18—C9—C19—C13	0.9 (2)
C6—C7—O8—C18	−0.8 (2)	C4—C13—C19—O1	179.76 (12)
O4—C4—C13—C19	177.50 (13)	C3—C13—C19—O1	0.98 (16)
C14—C4—C13—C19	−1.4 (2)	C4—C13—C19—C9	0.0 (2)
O4—C4—C13—C3	−4.3 (3)	C3—C13—C19—C9	−178.78 (14)
C14—C4—C13—C3	176.88 (16)	C13—C4—O4—C41	−3.8 (2)
C2—C3—C13—C4	−179.08 (17)	C14—C4—O4—C41	175.09 (12)
C2—C3—C13—C19	−0.70 (16)	C4—O4—C41—C42	−166.21 (12)
O4—C4—C14—C18	−177.18 (12)	O4—C41—C42—C43	141.31 (15)
C13—C4—C14—C18	1.8 (2)	C41—C42—C43—C49	2.1 (2)
O4—C4—C14—C5	3.7 (2)	C41—C42—C43—C44	−176.19 (14)
C13—C4—C14—C5	−177.31 (13)	C42—C43—C44—C45	113.54 (16)
C6—C5—C14—C18	0.8 (2)	C49—C43—C44—C45	−64.89 (17)
C6—C5—C14—C4	179.90 (14)	C43—C44—C45—O5	−53.12 (17)
C19—C9—C18—O8	179.60 (12)	C43—C44—C45—C46	−171.68 (13)
C19—C9—C18—C14	−0.4 (2)	O5—C45—C46—C47	109.83 (18)
C7—O8—C18—C9	−178.14 (13)	C44—C45—C46—C47	−128.43 (17)
C7—O8—C18—C14	1.8 (2)	C45—C46—C47—C50	−0.7 (3)
C4—C14—C18—C9	−0.9 (2)	C45—C46—C47—C48	179.69 (15)
C5—C14—C18—C9	178.22 (14)		

### Hydrogen-bond geometry (Å, °)

**Table:**

*D*—H···*A*	*D*—H	H···*A*	*D*···*A*	*D*—H···*A*
O5—H51···O8^i^	0.84	2.57	3.2193 (16)	135

Symmetry codes: (i) −*x*+1, −*y*+2, −*z*+1.
